# The role of long non-coding RNA in hepatocellular carcinoma

**DOI:** 10.18632/aging.205523

**Published:** 2024-02-08

**Authors:** Weizheng Liang, Yan Zhao, Qingxue Meng, Wenjie Jiang, Shoulong Deng, Jun Xue

**Affiliations:** 1 Central Laboratory, The First Affiliated Hospital of Hebei North University, Zhangjiakou 075000, Hebei, China; 2 Department of General Surgery, The First Affiliated Hospital of Hebei North University, Zhangjiakou 075000, Hebei, China; 3 Tumor Research Institute, The First Affiliated Hospital of Hebei North University, Zhangjiakou 075000, Hebei, China; 4 Department of Mathematics and Computer Science, Free University Berlin, Berlin 14195, Germany; 5 Technology Department, The First Affiliated Hospital of Hebei North University, Zhangjiakou 075000, Hebei, China; 6 Department of Artificial Intelligence and Data Science, Hebei University of Technology, Tianjin 300401, China; 7 National Health Commission of China (NHC) Key Laboratory of Human Disease Comparative Medicine, Institute of Laboratory Animal Sciences, Chinese Academy of Medical Sciences and Comparative Medicine Center, Peking Union Medical College, Beijing 100021, China

**Keywords:** hepatocellular carcinoma, long non-coding RNA, prognosis

## Abstract

Hepatocellular carcinoma (HCC) is a prevalent liver malignancy with complex etiology and generally poor prognosis. Recently, long non-coding RNAs (lncRNAs), non-protein-coding RNA molecules exceeding 200 nucleotides, have emerged as pivotal players in HCC, influencing its initiation, progression, invasion, and metastasis. These lncRNAs modulate gene expression at epigenetic, transcriptional, and post-transcriptional levels, actively participating in the pathological and physiological processes of HCC. Understanding the intricate relationship between lncRNAs and HCC is important for improving prognosis and reducing mortality. This review summarizes advancements in elucidating the role of lncRNAs in HCC pathogenesis.

## INTRODUCTION

Non-coding RNAs (ncRNAs) refer to functional RNA molecules that are not translated into proteins, such as ribosome RNA (rRNA) transcribed by RNA polymerase I, transfer RNA (tRNA) transcribed by RNA polymerase III, microRNA (miRNA) transcribed by RNA polymerase II and so on [1, 2]. In recent years, accumulating studies have demonstrated that ncRNAs play important roles in biological epigenetics by regulating gene expression at both the transcriptional and post-transcriptional levels. Those RNA molecules are considered as RNA genes. Generally, ncRNAs can be divided into several types, including tRNA, rRNA, small RNAs (such as miRNAs and siRNAs and long non-coding RNAs (lncRNAs) based on their lengths [3, 4]. LncRNAs normally refer to RNA molecules with a length of more than 200 nt that lack the ability to encode proteins. In this review, we mainly focus on lncRNAs and their functionalities involved in Hepatocellular carcinoma (HCC).

HCC is the most common pathological type of primary liver cancer which accounts for 75% to 85% of liver cancer cases. The latest edition of the cancer statistics of the United States in 2023 showed that HCC is the sixth most frequently diagnosed cancer in the world and has surpassed gastric cancer to become the third leading cause of cancer death. There were about 41,210 new cases and 29,380 deaths from Liver and intrahepatic bile duct concerned cancers around the U.S in 2023 [5]. The vital reasons for the poor prognosis of patients with HCC include: the symptoms and signs in the early stage are not easily noticed by patients, lacking effective treatment methods, and frequent tumor metastasis [6]. Moreover, due to limitation of therapeutic options, and high mortality rates, HCC stands as a formidable challenge for researchers and clinicians. In this landscape of medical urgency, understanding the intricate molecular mechanisms that underlie HCC becomes not only significant but imperative. It is important to explore novel tumor markers and therapeutic targets to improve the prognosis of HCC patients.

In recent decades, research on the molecular mechanism of HCC has mainly focused on the protein-encoding oncogenes and tumor suppressor genes. Recently, the development of deep sequencing technologies has made scientists turn their attention to ncRNAs including lncRNAs. Some recent studies have found that the abnormal expression of lncRNA is closely related to the occurrence and development of tumors, indicating that lncRNAs play vital roles in the early diagnosis and clinical treatment of malignant tumors. For HCC, lncRNAs have been reported to be related to several types of biological behaviors, such as tumor angiogenesis, cell proliferation, vascular invasion, and metastasis [7–20]. Furthermore, the expression levels of classic lncRNAs have been found to be significantly dysregulated in tumor tissues compared with normal liver tissues around [21]. In this review, we thoroughly summarized the up-to-date research progress of lncRNAs in HCC and highlighted the functions of lncRNAs in the pathogenesis of HCC to throw light on follow-up research in this field.

## Overview of lncRNAs

**Figure 1:**
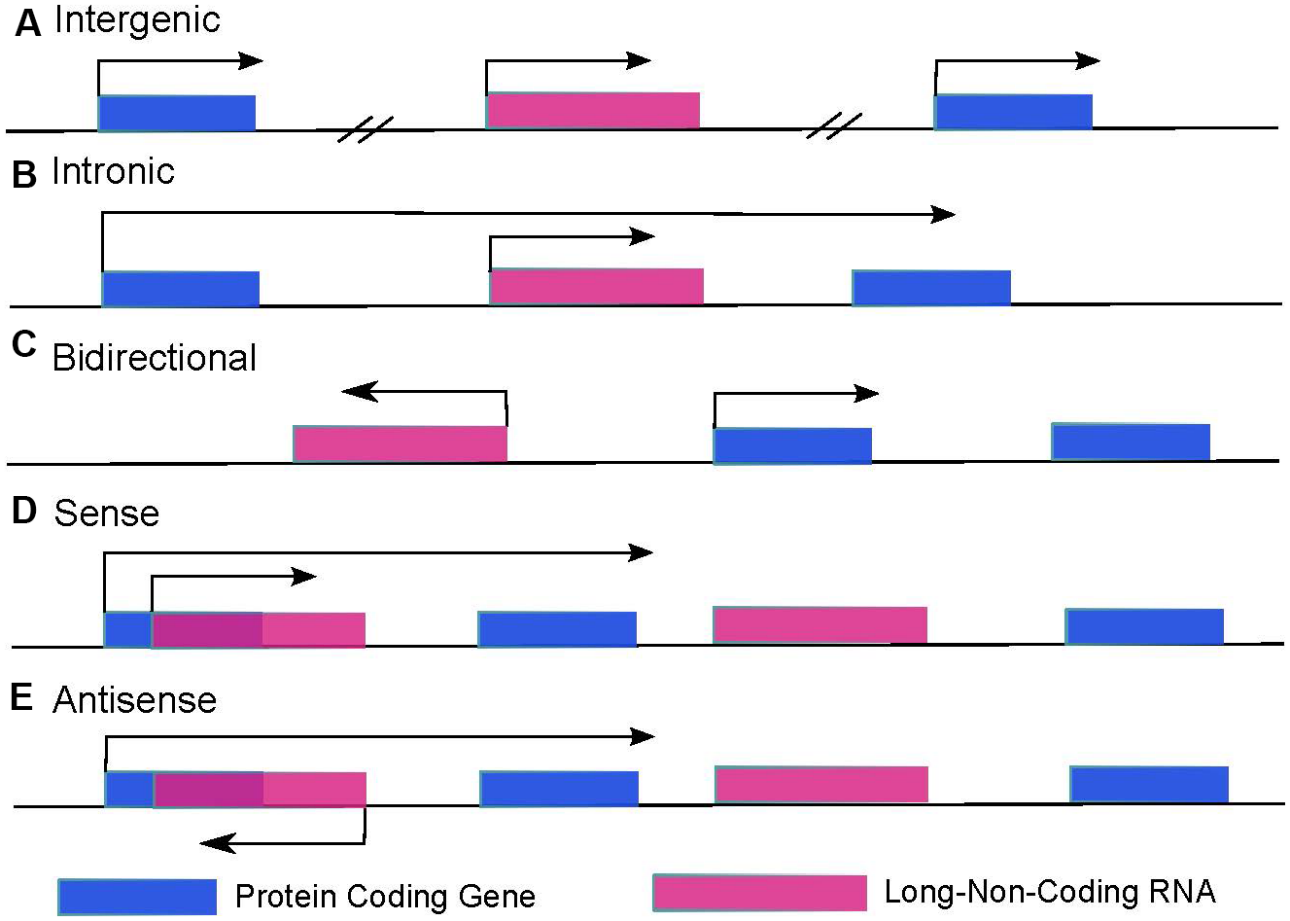
**LncRNAs categories are decided by the location of lncRNAs and surrounding protein-coding genes.** (**A**) Intergenic lncRNAs are autonomously transcribed non-coding RNAs longer than 200 nucleotides that do not overlap annotated coding genes. (**B**) Intronic lncRNAs are lncRNAs that locate inside of an intron of a protein-coding gene and can initiate in either direction. (**C**) Bidirectional lncRNAs are transcripts that are transcribed from the same promoter as a protein-coding gene but in the opposite direction. (**D**) Sense lncRNAs overlap with one or more introns and/or exons of a protein-coding gene in the sense direction. (**E**) Antisense lncRNAs are transcribed from the opposite strand of protein-coding genes.

In the human genome, only about 2% of regions can be finally translated into proteins after transcription, and the remaining 98% of regions are supposed not to encode any proteins. The transcription products that cannot be translated into proteins are defined as non-coding RNAs, normally featured with a high density of terminators and lacking effective open reading frames. In the past, ncRNAs were once thought to be noisily produced during transcription (junk RNA), but growing evidence suggests that ncRNAs are not genomic junk. Instead, they are functional RNA molecules. Many different kinds of ncRNAs such as miRNA, snoRNA, piR-NA, and lncRNA were found to be functional in many transcriptional and post-transcriptional processes [22]. It has been reported that the majority of ncRNAs are lncRNAs, the length of which is generally more than 200 nt, and their open reading frames are generally within 50~100 nt [23]. According to the source of their transcription position in the genome, lncRNA can be divided into long intergenic non-coding RNA (lincRNA, Figure 1A), intron-derived long non-coding RNA (Figure 1B), lncRNA transcribed against mRNA direction (bidirectional lncRNA, Figure 1C) and mRNA sense (Figure 1D) and antisense strand RNA (natural antisense transcript, NAT, Figure 1E), etc. [24].

**Figure 2:**
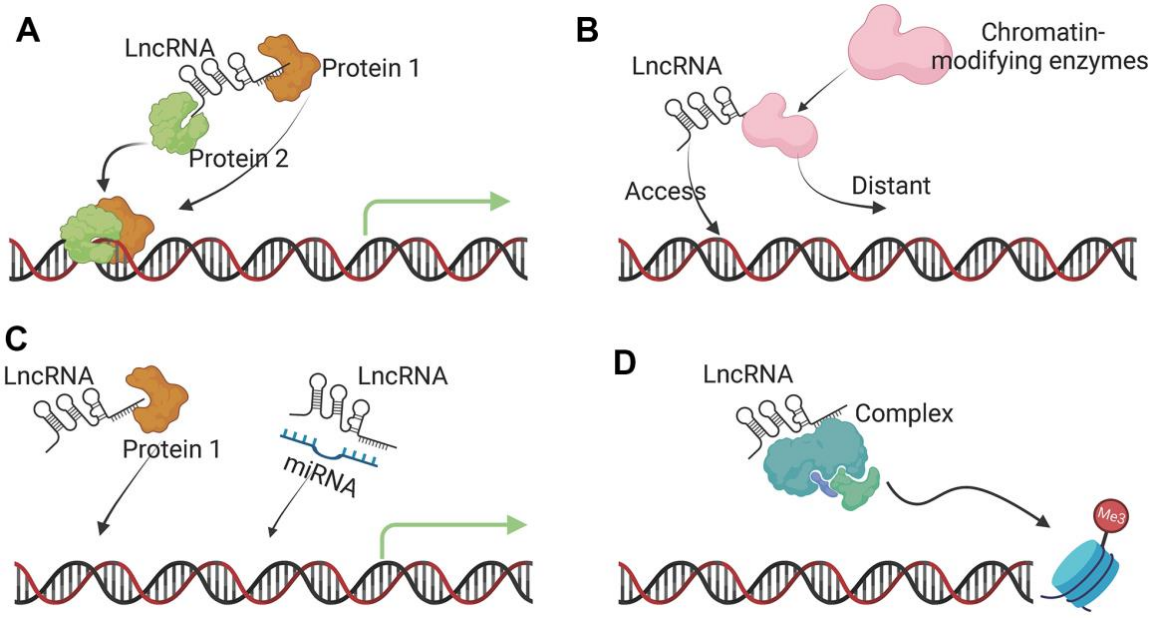
**Molecular functions of lncRNAs.** (**A**) Signal lncRNA. The main function of a signal lncRNA is to serve as a molecular signal to regulate the transcription of certain genes in response to various stimuli, such as transcription factors or chromatin modifiers. (**B**) Guide lncRNA. Guide lncRNAs interact with chromatin-modifying enzymes and direct them to the specific genomic location to regulate gene expression. (**C**) Decoy lncRNA. Decoy lncRNAs bind to microRNAs or transcription factors to sequester them away from their targets, affecting transcription and translation. (**D**) Scaffold lncRNA. lncRNAs can act as dynamic scaffolds for multiple-component complexes, such as ribonucleoprotein (RNP) complexes to transiently assemble and affect histone modifications.

**Figure 3:**
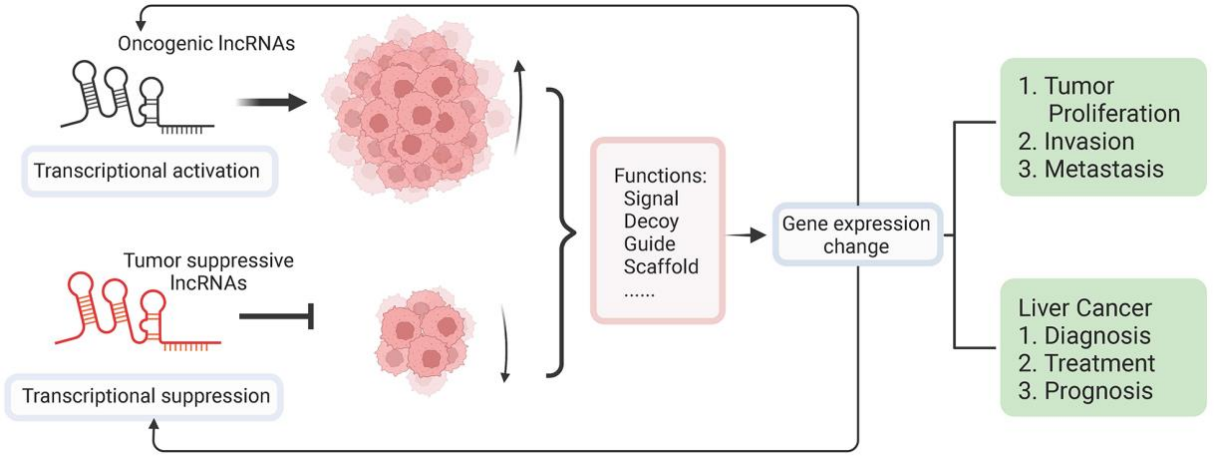
**Overview of molecular functions of lncRNA in liver cancer and its application in clinical treatments.** LncRNAs dysregulation as a result of epigenetic and genetic alterations makes lncRNAs perform as either activators or suppressors during the initiation of liver cancer. Functioning as signal, guide, decoy, and scaffold, the dysregulated expression of lncRNAs can affect the expression of protein-coding genes, which further induce complicated interactions and regulations resulting in positive or negative affection on tumor proliferation and metastasis. The dysregulated lncRNAs could be used as markers for diagnosis, targets of treatments, and potential indicators of the prognosis of liver cancer.

The main regulatory function of lncRNA is to interact with DNA, proteins, and other RNA molecules. In cells, lncRNAs regulate gene expression by extensively regulating DNA methylation, histone modifications, and chromosomal remodeling. In the transcription process of eukaryotic species, several proteins like transcription factors and RNA polymerase II are required to form a transcription initiation complex. Similar to the expression of protein coding genes, LncRNAs are mainly transcribed and spliced by RNA polymerase II [25]. LncRNAs then affect the transcription and translation of DNA through a series of cis- and trans-regulations. In addition, lncRNAs can also complementarily pair with target mRNA sequences, thereby affecting the process of mRNA splicing, editing, transport, translation, and degradation [26–28]. To sum up, the main functions of lncRNA include: (1) serving as a molecular signal to regulate the transcription of certain genes in response to various stimuli, such as transcription factors or chromatin modifiers (Figure 2A); (2) acting as a guide to mediate the binding of histone modification complexes to chromatin (Figure 2B); (3) competitive binding with miRNAs to weaken their regulatory ability (Figure 2C); (4) acting as a scaffolding molecule to mediate the formation of protein complexes (Figure 2D) [29, 30].

Many studies have reported that lncRNAs are related to many kinds of tumors in many different ways. Abnormal expression is one of the ways lncRNAs play roles in several tumor types, including breast cancer, gastric cancer, liver cancer, colorectal cancer, and cervical cancer [31–34]. The lncRNAs are closely related to the occurrence and development of tumors, including excessive tumor cell proliferation, tumor suppressor gene inactivation, tumor cell immortalization, tumor metastasis and infiltration, and apoptosis inhibition [23, 35–37]. As for HCC, it has been shown that lncRNAs are involved in the occurrence, development, and clinical prognosis of HCC.

## LncRNAs associated with hepatitis-induced HCC occurrence

Chronic viral hepatitis, particularly hepatitis B (HBV) and C (HCV), is a major risk factor for the development of HCC. These viruses can cause prolonged liver inflammation, which increases the likelihood of genetic mutations in liver cells and the subsequent development of cancer. Over time, the liver damage caused by hepatitis can lead to cirrhosis, which is characterized by the replacement of healthy liver tissue with fibrous scar tissue. Liver cirrhosis significantly elevates the risk of developing HCC. Long non-coding RNAs (lncRNAs) have been shown to play a role in regulating the inflammatory response in the context of hepatitis.

HBx-LncRNA is an lncRNA associated with the hepatitis B x (HBx) protein which is a key regulatory protein produced by HBV. It plays a role in promoting the development of HCC by modulating various cellular processes, including cell proliferation, apoptosis, and immune responses. It is considered a potential oncogene in the context of HBV-related HCC. There have been many lncRNAs reported to be significantly upregulated in HBV infected patients and HCC patients. The lncRNAs include HBV Enhancer-Induced lncRNA (HEIH), HCC Up-Regulated Long Non-Coding RNA (HULC), Metastasis-Associated Lung Adenocarcinoma Transcript 1 (MALAT1), UC001kfo.1, Dreh, Upregulation of these lncRNAs promotes the progression of HCC for HBV infected patients.

There are also some lncRNAs which are significantly down-regulated in HCC. MEG3 is a tumor-suppressive lncRNA that is downregulated in HCC. It plays a role in inhibiting cell growth and promoting apoptosis. In the context of HBV-associated HCC, reduced MEG3 expression has been observed. LncRNA-p21 is another lncRNA that has been found to be downregulated in HCC tissues. Its downregulation is associated with increased cell proliferation and tumor growth. LncRNA-p21 can interact with various proteins and signaling pathways to inhibit HCC progression. For example, it can interact with p53, a well-known tumor suppressor gene, to enhance its activity. This interaction helps in controlling cell cycle arrest and preventing uncontrolled cell growth.

## Effects of lncRNAs in HCC on cell growth and proliferation

HCC Up-Regulated Long Non-Coding (HULC) RNA, which is located at 6p24.3 in the genome with a length of 500 nt, is the first abnormally highly expressed lncRNA observed in human HCC specimens [38]. In 2013, Hui Xie et al. investigated the expression levels of HULC in the plasma of 30 HCC patients and 20 healthy persons respectively. The investigation results showed that the detection rate of HULC in the plasma of HCC patients is relatively higher than that in the healthy group, indicating it can be considered as a novel plasma tumor marker for HCC [39]. In addition, the up-regulation of HULC was also observed in HCC tissues. The abundance of HULC in both tissue and plasma is positively correlated with Edmondson grade and hepatitis B virus infection. After two years, Jun Li et al. pointed out that both HULC and Linc00152 are significantly highly expressed in the plasma of HCC patients [40]. HULC is further reported its ability to affect the angiogenesis rate of HCC tumor tissue by up-regulating sphingosine kinase 1 (SPHK1), which ultimately promotes the continuous deterioration of HCC [41]. Several studies demonstrated that ectopic HULC decreased the expression of P62, and excessive HULC can increase the expression of LC3 at the transcriptional level, then activate LC3 through Sirt1 (deacetylase). Thus, the expression of autophagy-related genes (becline-1) is increased, which finally accelerates the malignant progression of hepatoma cells [42]. In addition, HULC can act as a competing endogenous RNA (ceRNA) to adsorb and inhibit the activity of a variety of miRNAs, including binding to miRNA-372, reducing the inhibitory effect of miRNA-372 on the target gene PRKACB (a catalytic subunit of cAMP-dependent protein kinase), thereby allowing translocation of PRKACB to the cell nucleus and the promoting phosphorylation of CREB. The phosphorylated transcription factor CREB further regulates the expression of HULC and increases its expression level, which is a positive feedback self-regulation process [43]. The interaction between HULC and miRNAs and the self-regulation process further contributes to the growth and proliferation of HCC cells. Yumei Du et al. have shown that the expression level of HULC in HBV-HCC tissue is positively correlated with HBx, which has a pivotal function in the occurrence and growth of HCC. Meanwhile, *in vitro* experiments have proved that CREB can up-regulate the expression of HULC, which inhibits p18 to promote the proliferation of hepatoma cells [44]. In addition, Imad J Matouk et al. have shown that HULC excessive expression can also be detected in colon cancer with liver metastases, while not detected in primary colon cancer, suggesting that HULC abnormal expression may be related to the internal environment of the liver [45].

Another lncRNA that is associated with HCC cell proliferation is lncRNA UFC1 (Ubiquitin-Fold Modifier Conjugating Enzyme 1), which is a target gene of miR-34a and can promote hepatoma cell proliferation, induce cell cycle, and inhibit cell apoptosis [46]. For UFC1-overexpressing HCC cells, the upregulation of miR-34a can effectively inhibit cell-cycle related proteins translation, cell proliferation, and HuR expression [47]. The function of UFC1 provides us with a new idea to develop it as a molecule therapy approach. Moreover, lncRNA hPVT1 also functions in promoting hepatoma cell proliferation and regulating cell cycle. The biological function of lncRNA hPVT1 depends on the presence of Nop2, which can effectively upregulate nucleolar protein P120 by increasing the stability of the Nop2 protein [48]. Previous studies have shown that TGF-1/lncRNA hPVT1/Nop2 signaling pathway is related to the progression of HCC. The hPVT1 can stimulate the stem cell-like potential of hepatoma cells to promote the growth of the hepatoma [48]. Interference of lncRNA hPVT1/Nop2 signaling pathway would be feasible in the treatment of HCC. LncRNA CCAT1 (Colon Cancer Associated Transcript 1) also correlates with cell proliferation. One early study confirmed that CCAT1 can upregulate the expression of c-Myc gene, thereby promoting the tumorigenesis [49]. CCAT1 inhibits the complete or incomplete complementary binding of Let-7 to target genes, resulting in the down-regulation of endogenous c-Myc and promoting cell proliferation and metastasis. For HCC cells, CCAT1 acts as a molecular sponge and then binds to HMGA2 and c-Myc, both of which are endogenous targets of the molecule Let-7. The inhibitory effect of the Let-7 results in enhanced proliferation and migration of HCC cells [50]. The studies indicate that CCAT1 and let-7 may be helpful in the early detection of HCC. Additionally, this study found that CCAT1 and let-7 could be related to the migration of HCC cells, providing potential therapeutic targets. Studies have found that serum linc-ITGB1 levels are significantly correlated with tumor size and distant tumor metastasis in HCC. Afterwards, it was proved by experiments that linc-ITGB1 overexpression can effectively promote the proliferation, migration, and invasion of HCC cells, as well as the expression of ROCK1. Meanwhile, the ROCK1 inhibitor can reduce the effect of linc-ITGB1 overexpression on cell migration, invasion, and proliferation [51–53].

The role of the lncRNA myocardial infarction-associated transcript (MIAT) is reported to be associated with micro-RNA, a regulatory molecule, in HCC cells (miR)-214 [54]. It is reported that MIAT was upregulated in HCC tissues and cells and contributed to HCC cell proliferation and invasion. MIAT achieved this by sponging micro-RNA (miR)-214, inhibiting miR-214 counteracted the effects of MIAT downregulation on cell proliferation and invasion. Moreover, in a mouse model, reducing MIAT levels significantly suppressed HCC tumor growth through the release of miR-214. This research provides novel insights into the potential therapeutic and prognostic significance of the lncRNA MIAT/miR-214 axis in HCC.

lncRNA FIRRE (Firre Intergenic Repeating RNA Element) is another over-expressed lnRNA in HCC tissues and cells, especially in cases with advanced clinical features. High FIRRE levels are associated with worse survival outcomes in HCC patients [55]. Functionally, reducing FIRRE levels suppresses HCC cell proliferation and glycolysis, while overexpression of FIRRE has the opposite effect. FIRRE also positively regulates the expression of the glycolytic enzyme PFKFB4 in HCC cells, which is linked to high tumor grade and advanced disease stage. PFKFB4 expression is associated with poor prognosis in HCC. Mechanistically, FIRRE acts in the cell nucleus and promoted PFKFB4 transcription and expression through cAMP-responsive element-binding protein (CREB). PFKFB4 could counteract the effects of FIRRE knockdown on HCC cell proliferation and glycolysis. In summary, FIRRE played a crucial role in promoting HCC cell proliferation and glycolysis by enhancing CREB-mediated PFKFB4 expression.

Another lncRNA UCA1 (Urothelial Cancer Associated 1) is located at human chromosome 19p13.12 with a length of 1439 nt. One study reported that UCA1 can activate the expression of the transcription factor Snail2 by adsorbing miR-203, thereby promoting the epithelial-mesenchymal transition (EMT) process, subsequently promoting the proliferation of HCC cells [56]. EMT is a biological phenomenon that permits a polarized epithelial cell, typically engaged with the basement membrane through its basal surface, to undergo a series of biochemical alterations, ultimately leading it to adopt a mesenchymal cell phenotype characterized by an increased migratory capacity [57].

MEG3, another important lncRNA located at chromosome 14q32 and expressed in most normal cells, is a maternally imprinted gene and a homolog of mouse maternally imprinted gene Gtl2. MEG3 acts as a tumor suppressor and is downregulated in many human tumors [58]. C. Braconi, et al. has found that MEG3 undergoes an expression inhibition in HCC which is caused by epigenetic regulation. There is CpG hypermethylation in the CRE region of the MEG3 promoter in HCC cells, which affects the transcription of MEG3 to be down-regulated by 210-fold in HCC cells compared with non-malignant hepatocytes, and MEG3 can be used as a target molecule of miRNA-29 to regulate the growth of HCC cells [59]. Yunli Zhou et al. have shown that MEG3 exerts tumor suppressor effects through both p53-dependent and p53-independent pathways in HCC [60]. MEG3 has the capability to diminish the expression of E3 ubiquitin ligase MDM2. This action leads to a reduction in the ubiquitination of p53 and an elevation in p53 protein levels. As a result, p53’s enhanced presence contributes to its increased binding to the promoter region of the cell growth inhibitor GDF15, consequently promoting the transcription of GDF15. This, in turn, hinders the occurrence of HCC. The study further found that MEG3 can also inhibit the proliferation of p53 knock-out hepatoma cells. Therefore, MEG3 may exert a tumor suppressor effect through other pathways [60].

In addition to several lncRNAs mentioned above, many other lncRNAs have also been identified to participate in the regulation of hepatoma liver cancer cell proliferation. The lncRNA GIHCG (GIHCG Inhibitor of MiR-200b/200a/429 Expression) promotes the proliferation and metastasis of hepatoma cells by increasing histone H3K27 methylation in the promoter region of miR-200b/a/429, thereby silencing the expression of miR-200b/a/429 [61]. Chen et al. found that another lncRNA, NR027113, was significantly up-regulated in HCC samples. They deduced that NR027113 could promote the proliferation and invasion of HCC through PTEN/PI3K/AKT signaling pathway, and suggested it could be a potential therapeutic target for HCC treatment [62]. HOTAIR (HOX Transcript Antisense RNA) which was found in the HOXC gene on chromosome 12 of the human genome is the first lncRNA found to have a reverse transcription [63–65]. HOTAIR is highly expressed in HCC tissue, and it regulates glucose metabolism by activating the mTOR signal transduction pathway, thereby promoting the cell proliferation [66]. It’s also reported that the growth of HCC cells is significantly inhibited by knocking down HOTAIR in the HCC cell line HepG2 [67]. Further studies showed that HOTAIR could regulate the expression of OGFr. Down-regulation of HOTAIR can induce the expression of OGFr at mRNA and protein levels. The results of CCK-8 experiments showed that OGFr knockdown significantly promoted the proliferation of HCC cells, while overexpression of OGFr did not significantly reduce cell proliferation, indicating that HOTAIR acts as a positive regulator of HCC cell proliferation. While OGFr is a negative regulator of HCC cell proliferation, HOTAIR may affect HCC cell proliferation by regulating the expression level of OGFr. PTTG3P (Pituitary Tumor-Transforming 3) is an intron-less gene that is highly homologous to its family members PTTG1 and PTTG2. Huang et al. found that lncRNA PTTG3P can promote the expression of PTTG1 followed by activating the Pl3K/AKT signaling pathway, then upregulating C-myc and cyclinD1, increasing the phosphorylation level of Rb protein, and finally promoting the proliferation of HCC cells [68]. In HBV-related HCC, DREH is negatively correlated with the expression of HBx. Inhibition of DREH can promote HBx-induced HCC cell proliferation [69]. Therefore, the regulation of DREH on HBV-related HCC cell proliferation may become a potential target for the prevention and treatment of HBV-related HCC. LincUSP16, as a ceRNA of miR-21 and miR-590-5p, can increase the expression of PTEN, which in turn leads to the inhibition of the AKT pathway and the inhibition of HCC cell proliferation. Thus, it can be concluded that lincUSP16 functions as a tumor suppressor in the development of HCC. The related signaling pathway could be used for the treatment of HCC [70].

In sum, various lncRNAs (as listed in Table 1 as well) have promoting or suppressing effects on cell proliferation and growth in HCC, which are worth being paid attention to in the future as potential therapeutic targets. Meanwhile, novel lncRNA candidates need to be investigated to deepen our understanding of the mechanisms of HCC.

**Table 1:** Summary of the roles and mechanisms of lncRNAs on cell growth and proliferation in HCC.

**lncRNA**	**Role in HCC**	**Mechanisms**
HULC	Highly expressed in HCC specimens	Up-regulates SPHK1, affects angiogenesis, regulates autophagy, functions as a ceRNA to modulate miRNA activity
UFC1	Promotes HCC cell proliferation	Target gene of miR-34a, regulates cell cycle, inhibits cell apoptosis
hPVT1	Promotes cell proliferation and cell cycle	Regulated by Nop2, stimulates stem cell-like potential, related to TGF-1 pathway in HCC
CCAT1	Upregulates c-Myc, promotes tumorigenesis	Acts as a molecular sponge for Let-7, enhances cell proliferation and migration
ITGB1	Promotes proliferation, migration, invasion	Correlated with tumor size and metastasis, overexpression promotes HCC cell characteristics
UCA1	Promotes proliferation via EMT	Activates Snail2 via miR-203, promotes EMT, subsequent proliferation of HCC cells
MEG3	Tumor suppressor, downregulated in HCC	Regulated by miRNA-29, affects p53-dependent and p53-independent pathways, inhibits cell proliferation, potential target of miRNA-29
GIHCG	Promotes proliferation and metastasis	Upregulates histone methylation in miR-200b/a/429 promoter region, silencing miR-200b/a/429 expression
NR027113	Promotes proliferation and invasion	Upregulated in HCC, modulates PTEN/PI3K/AKT signaling, potential therapeutic target
HOTAIR	Promotes glucose metabolism, proliferation	Regulates mTOR pathway, affects cell proliferation, related to OGFr and p53, affects HCC cell proliferation
PTTG3P	Promotes proliferation via AKT pathway	Upregulates PTTG1, activates PI3K/AKT, promotes cell-cycle-related proteins, C-myc and cyclinD1 expression, promotes HCC cell proliferation
DREH	Inhibits HBx-induced cell proliferation	Negatively correlated with HBx, potential target for prevention and treatment of HBV-related HCC
LincUSP16	Functions as a tumor suppressor	Binds to miR-21 and miR-590-5p, increases PTEN expression, inhibits AKT pathway, inhibits HCC cell proliferation

## Roles of lncRNAs in HCC on cell invasion and metastasis

Invasion and metastasis of HCC are vital factors affecting the therapeutic effects on HCC patients. It has been found that various lncRNAs are involved in the process mainly by affecting the EMT, targeting miRNA, regulating signaling pathways, and promoting angiogenesis. The function of this lncRNAs will be discussed in this section, and a brief summary of the roles and function mechanisms of these lncRNAs can be found from Table 2.

**Table 2:** Summary of the roles and mechanisms of lncRNAs in HCC on cell invasion and metastasis.

**lncRNA**	**Role in HCC metastasis and invasion**	**Mechanisms and pathways involved**
H19	Both tumor-promoting and suppressing roles, dysregulated epigenetic expression	Upstream regulatory regions, downstream pathways, tumor-promotion in HCC tissues
MALAT1	Promotes angiogenesis, accelerates HCC development and metastasis	MALAT1/miR-140/VEGF-A axis, interaction with miR-204, regulation of SIRT1, linked to HCC recurrence
HEIH	High expression in HCC, related to HBV-related HCC progression	Inhibition of cell differentiation, associated with EZH2 gene, prognostic indicator for HCC survival
ATB	Highly expressed in multiple cancers, promotes tumor metastasis	Binds to miR-200 family, promotes ZEB1 and ZEB2 expression, enhances invasive ability
LET	Tumor suppressor, associated with hypoxia-induced cancer metastasis	Inhibits LET expression, affects NF90 downstream genes, enhances hepatocyte metastasis
HOTTIP	Tumor-promoting, enhances distant metastasis of HCC	Regulates miRNA-125b, influences miRNA-125b expression, promotes HCC progression
CCAT2	Regulates EMT and metastasis, promotes HCC growth and development	Upregulates FOXM1, competitive binding to miR-34a, enhancement of HCC cell proliferation
SPRY4-IT1	Promotes EMT, enhances growth and invasive ability of HCC	Up-regulates Twist1 and Vimentin, inhibits E-cadherin transcription, TNF signaling pathway involvement
AOC4P	Tumor suppressor, inhibits EMT, reduces proliferation, invasion, metastasis of HCC	Upregulated expression inhibits EMT, binding to Vimentin protein, reduces invasion, metastasis
CAR-Lo-5	Enhances HCC infiltration and invasion, regulates miRNA-200b, associated with poor prognosis	Promotes EMT, silencing of miRNA-200b, influences ZEB1 and ZEB2 protein levels, promotes invasion
CASC2	Tumor suppressor, inhibits EMT, promotes apoptosis, reduces metastasis of HCC	Inhibition of MAPK signaling pathway, involvement in CASC2/miRNA-367/FBXW7 axis, reduction of lung metastasis

H19 is a maternally imprinted gene located at human chromosome 11p 15.5, while IGF2 is a paternally imprinted gene similar to H19 in localization. Dysregulated epigenetic expression of the two genes is detected in HCC cells [71]. H19 can exert opposite effects of tumor-promoting or tumor-suppressing through different molecular mechanisms in HCC, which are related to different upstream regulatory regions and downstream pathways. Previous studies have found that H19 is a tumor-promoting factor in HCC cells. The expression level of H19 in HCC tissues is significantly higher than that in normal tissues. Knockout of the H19 gene leads to significantly reduced proliferation and invasion ability of HCC cells. Aflatoxin B1 promotes the proliferation and invasion of hepatoma cells by increasing the expression levels of H19 and transcription factor E2F1 [72]. H19 inhibits HCC cell migration by Hnrnpu/PCAF/RNA Pol-II or activates the miR-200 family by increasing histone acetylation, thereby inhibiting HCC metastasis [73]. In addition, as a downstream target of the AKT/GSK3T/GSK25A signaling pathway, H19 influences the invasion and metastasis of the HCC [74]. H19 can also promote cell proliferation, invasion, and migration by activating the CDC42/PAK1 signaling pathway in HCC through targeting miR-15b [75].

LncRNA MALAT1 (Metastasis Associated Lung Adenocarcinoma Transcript 1) was firstly identified in non-small cell lung cancer. The MALAT1 gene is located at chromosome 11q13.1, about 6.7kb in length, and is ubiquitously expressed in human and mouse cells [76]. Studies have shown that LncRNA MALAT1 is associated with the progression of malignant tumors in tissues such as the liver, pancreas, lung, and bladder, and is considered to be an oncogene-like lncRNA. MALAT1 is a key regulator in HCC and is closely related to tumor metastasis and recurrence. It has been revealed that MALAT1 can promote angiogenesis, thereby accelerating the development and metastasis of HCC, indicating that targeting the MALAT1/miR-140/VEGF-A axis may be an effective way to treat HCC in the future [77]. Another study found that expression of MALAT1 was increased in HCC cells, and the content of miR-NA-204 was significantly down-regulated in sh-MALAT1 HepG2 cells and 15 HCC tissues, suggesting miR-NA-204 may act as a potential interaction partner of MALAT1. At the same time, SIRT1 is the direct downstream target of miRNA-204 in HepG2 cells. Therefore, overexpression of MALAT1 reduces the expression of miRNA-204 through negative regulation, thereby silencing the expression of SIRT1, and promoting the invasion and metastasis of the HCC [78]. Lai et al. found that high expression of MALAT1 significantly increased the risk of HCC recurrence in liver transplant patients [79]. In summary, it is shown that MALAT1 is closely related to the invasion and metastasis of HCC. The expression of MALAT1 can be used as an independent factor to predict the recurrence and metastasis of HCC, which may shed light on a novel target for the treatment of recurrence and metastasis of HCC.

LncRNA HEIH (Hepatocellular Carcinoma Up-Regulated EZH2-Associated Long Non-Coding RNA) has high expression in HCC, which is related to the progression of HBV-related HCC, and its expression in HCC tissue is higher than that in non-cancer liver tissues. Studies have found that HEIH can block the G0/G1 phase of cell differentiation and is related to the EZH2 gene. EZH2 is the core catalytic factor of PRC2, which can inhibit the transcription of target genes by methylating histones to promote the occurrence and development of HCC [80]. Patients with high HEIH expression have a worse prognosis than the ones with low HEIH expression. High HEIH expression is associated with HCC recurrence. HEIH can be used as a prognostic indicator to evaluate the survival rate of HCC patients.

LncRNA ATB (LncRNA Activated By TGF-Beta), a newly discovered lncRNA, is located at chromosome 14 and is thought to be involved in the process of tumor progression and metastasis. Studies have demonstrated that ATB is highly expressed in colorectal cancer, renal cell carcinoma, and breast cancer, and is closely related to tumor microvascular and macrovascular metastasis and vascular invasion [81–86]. Meanwhile, ATB can combine with interleukin-11 (interleukin-11, IL-11) mRNA to promote the secretion of IL-11. In a nude mouse xenograft model, it was shown that IL-11 secreted by HCC cells contributes to distant colonization. Deletion of IL-11 in HCC cells attenuated the distant colonization of ATB, but the process did not induce or reverse the occurrence of EMT [87]. Therefore, ATB can regulate the progression of the EMT process affecting the local invasion of HCC, and it can also increase the probability of distant metastasis of HCC by promoting the secretion of IL-11.

LncRNA LET is a down regulated lncRNA in tumors, which functions as a tumor suppressor gene in normal tissues and is associated with hypoxia-induced cancer metastasis in cancer tissues. The hypoxic microenvironment formed by cancer tissue increases the level of histone deacetylase 3, which inhibits the expression of LET, thereby reducing the degradation of nuclear factor 90 (NF90), resulting in altered levels of NF90 downstream genes, such as HIF1 and other protein coding genes, thus promoting hepatocyte metastasis. *In vitro* and *in vivo* experiments show that LET can effectively inhibit the invasion and metastasis of HCC [88].

LncRNA HOTTIP (HOXA Distal Transcript Antisense RNA) was originally found in fibroblasts at the distal end of the body. The HOTTIP gene is located at the 5’ end of the HOXA gene cluster (chromosome 7p15.2), so it is called HOXA distal transcript, which belongs to the tumor-promoting gene lncRNA [89]. In a nude mouse transplantation model, it was found that by reducing HOTTIP expression, the ability of distant metastasis of HCC was significantly inhibited (especially in the lung) [90]. Further studies found that miRNA-125b acts as a post-transcriptional downstream target of HOTTIP in HCC cells, and HOTTIP overexpression may lead to loss of miRNA-125b expression, thereby enhancing the invasion and metastasis ability of HCC [90]. Therefore, HOTTIP may play an important role in promoting invasion and distant metastasis of HCC by regulating the expression of miRNA-125b.

LncRNA CCAT2 (Colon Cancer Associated Transcript 2) is located at the 8q24 region of the human genome and plays a role in tumor growth, metastasis, and destabilization of related chromosomal structures [91–94]. In HCC, people found that silencing CCAT2 in HCC can down-regulate the expression of vimentin and zinc finger transcription factor Slug (Snail2), and up-regulate the expression of E-cadherin, thereby inhibiting the EMT and metastasis [95]. In addition, other studies found that the forkhead box (FOX) transcription factor M1, a downstream target of the Wnt signaling pathway, can promote the expression of CCAT2; conversely, CCAT2 can also competitively bind to miR-34a and co-regulate the expression of FOXM1, thereby promoting the proliferation of HCC cells. This indicates that CCAT2 upregulates the expression of FOXM1 and promotes the occurrence and development of HCC [96, 97]. Another study found that high expression of CCAT2 is negatively correlated with the overall survival of intrahepatic cholangiocarcinoma (IHCC) patients [98]. *In vitro* experiments further confirmed that CCAT2 can promote proliferation, invasion, and metastasis of IHCC, indicating that CCAT2 can be used as an independent risk factor for poor prognosis in the IHCC [98]. All the above studies indicate that CCAT2 may become a promising target for HCC treatment.

LncRNA SPRY4-IT1 (SPRY4 Intronic Transcript 1) is located at the 5p31.3 region of the human genome. SPRY4-IT1 can affect a series of biological processes such as tumor cell proliferation and invasion by regulating cell cycle, RNA processing, and signaling molecule transmission [99–101]. One study showed that overexpressed SPRY4-IT1 can up-regulate the expression of Twist1 and Vimentin, and inhibit transcription of E-cadherin, thereby promoting the EMT process of HCC tissue, enhancing the growth and invasive ability of HCC, and increasing the probability of extrahepatic metastasis of HCC [102]. Similarly, another study also proved that SPRY4-IT1 has promoted effect on the metastasis of HCC, in which it was shown that SPRY4-IT1 could be involved in HCC cell proliferation, metastasis, and EMT and promotes HCC progression and metastasis through the TNF signaling pathway [103]. The above studies show that SPRY4-IT1 is closely related to the invasion and metastasis of HCC via multiple mechanisms. These findings are supposed to provide a novel target for the treatment of recurrence and metastasis of HCC in the future.

LncRNA AOC4P (Amine Oxidase Copper Containing 4) is a tumor suppressor gene located at the 17q21.31 region of the human genome. It has been found that AOC4P expression is significantly down-regulated in HCC tissues compared with adjacent normal tissues. Low AOC4P expression observed in clinical data also indicates poor prognosis in patients [104]. In addition, *in vitro*, functional analysis showed that high AOC4P expression could significantly reduce the proliferation, invasion, and metastasis of HCC cells by inhibiting the EMT process. *In vivo* xenograft experiments and tail vein migration experiments in nude mice showed that high expression of AOC4P can significantly reduce tumor growth rate and lung metastasis probability. Further exploration of the mechanism of lung metastasis proved that AOC4P may inhibit the process of EMT by binding to Vimentin protein and promoting its degradation, thereby inhibiting the invasion, progression, and distant metastasis of HCC.

LncRNA CAR-Lo-5 is in the 8q24 region of the human genome and is thought to account for cell cycle regulation and tumor development and is associated with the cancer susceptibility [105]. A previous study showed that overexpression of CAR-Lo-5 in HCC cells could enhance the infiltration and invasion ability of HCC. At the same time, the expression level of miRNA-200b decreased, showing a negative correlation with the expression of CAR-Lo-5. Further study suggested that CAR-Lo-5 and EZH2 can jointly silence the expression of miRNA-200b, thereby promoting the invasion and metastasis of the HCC [106]. People also found that up-regulation of CAR-Lo-5 in HepG2 HCC cells can significantly promote proliferation and migration of HCC, and abnormally high expression level of CAR-Lo-5 is associated with poor prognosis for HCC patients [107]. Therefore, the expression level of CAR-Lo-5 can be used as an independent factor to evaluate the prognosis of patients, and inhibiting the expression of CAR-Lo-5 provides a new idea for the prevention and treatment of HCC growth and metastasis.

LncRNA CASC2 (Cancer Susceptibility 2) is located at chromosome 10 of the human genome and plays a tumor suppressor role in the cancer [108, 109]. Palmieri et al. confirmed that CASC2 can inhibit EMT of HCC cells, achieve the purpose of inhibiting migration, invasion, and proliferation of HCC cells, and promote apoptosis of HCC cells by effectively inhibiting MAPK signaling pathway [109]. A previous study showed that the expression of CASC2 was reduced in HCC cells, and has an inhibitory function in invasion, metastasis, and EMT progression of HCC cells by participating in the CASC2/miRNA-367/FBXW7 axis [110]. Moreover, a mouse lung metastasis model showed that CASC2 overexpression significantly reduced lung metastasis probability in the primary HCC [110]. Another study showed that overexpression of CASC2 can reduce the ability of HCC invasion and metastasis, and promote apoptosis by inactivating the protein kinase signaling pathway, thereby reducing the metastasis ability of HCC cells [111]. In addition, CASC2 can downregulate miR-183 by inactivating the Wnt/β-catenin signaling pathway, thereby inhibiting migration and invasion abilities of HCC cells [112]. CASC2 can also regulate HCC cell activity through miR-362-5p/Nf-κB axis [113]. Therefore, CASC2 may reduce the incidence of distant metastasis of HCC through multiple signaling pathways, which provides the basis for designing therapeutic strategies to inhibit HCC growth and progression in the future.

## The role of LncRNA in the diagnosis, treatment, and prognosis of HCC

The prognosis of HCC is closely related to the clinical stage of the cancer [114]. Improving the early diagnosis rate of HCC can reduce the mortality of HCC and improve the prognosis of patients. Most early-stage HCCs have no obvious clinical symptoms. When clinical symptoms are found, tumor cells have often become invasive and metastasized, thus the clinical prognosis is often poor and the survival rate of patients is low. Based on current diagnostic methods, the diagnosis rate of early-stage HCC is not satisfying. Studies have shown that in patients with early-stage HCC, only 10% to 20% of patients have abnormal alpha-fetoprotein (AFP) [115, 116]. AFP was no longer used as a diagnostic indicator in the clinical guidelines since 2011 [117]. In recent years, although some new diagnostic indicators of HCC have been used in clinical practice such as abnormal prothrombin (DCP) to improve the diagnosis of HCC, these markers still have certain limitations. With the progress of lncRNA research, especially with deeper insight into the relationship between lncRNA and HCC, more and more lncRNAs have been explored and revealed to be useful for the diagnosis of early HCC.

SNPs of lncRNA may be associated with disease susceptibility. SNPs of ZNRD1 eQTLs increase the risk of HCC in HBV carriers but are protective against chronic HBV infection [118]. ZNRD1 antisense RNA 1 plays different roles in HBV-positive and HBV-negative HCC patients, resulting in different treatment options for these patients. Moreover, lncRNA can be applied to the diagnosis of HCC. Several studies have proved that UCA1 is a good indicator in diagnosing HCC. One study indicated that UCA1 has high specificity in the screening of HCC and it facilitate with distinguishing benign liver disease from HCC patients [119]. Another study got similar conclusions, in which researchers also explored the diagnostic value of UCA1 in distinguishing chronic hepatitis C patients from HCC patients and got similar conclusions. They found that UCA1 displayed a higher diagnostic efficacy with 0.838 AUC value [120]. LncRNA GAS5-AS1 is another potential diagnostic marker for HCC. One previous study showed that down-regulated GAS5-AS1 was associated with the occurrence of HCC. Further studies found that GAS5-AS1 had high accuracy in distinguishing HCC from cirrhosis (AUC = 0.824). When distinguishing HCC cases with AFP < 200 ng/ml from cirrhosis cases with AFP < 200 ng/ml as well as distinguishing HCC cases with AFP < 200 ng/ml and hepatitis B cases, GAS5-AS1 has a high sensitivity with 89.5% and 89.5%, respectively, indicating that GAS5-AS1 may be a potential diagnostic marker for HCC [121]. Additional research efforts have been put into investigating the possibility of other lncRNAs as diagnostic markers for HCC, including MALAT1, CASC9, SHNG1, Lnc-PCDH9-13:1, all of which have shown good diagnostic value [122–126]. While HCC diagnosis does not perform satisfying enough by using mono-lncRNA as an indicator, combining lncRNAs can often improve diagnostic efficiency. One study indicated that the combination containing 3 plasma lncRNAs LINC00152, RP11-160H22.5, and XLOC014172 may be effective to distinguish HCC patients from healthy people and chronic hepatitis patients [127]. Some other studies also confirmed the availability of multiple lncRNA as a potential diagnostic marker for HCC [128, 129]. In addition to the above implements mentioned, lncRNA can also be applied in the diagnosis of HCC by combining with AFP or other indicators [123, 130].

The prognostic Indicators of HCC are closely related to the expression levels of lncRNAs. One study analyzed the clinical data of 110 HCC patients after liver transplantation and found that the high expression of HOTAIR in HCC tissue can be used as an independent prognostic factor for HCC recurrence [131]. Another study also proved this (additional evidence is provided by Ishibashi M. et al.) [132]. Similarly, patients with high MALAT1 expression are very likely to recur after liver transplantation [79]. A study involving 102 HCC tissues and 21 normal liver tissues found that the overall survival (OS) (P=0.017) and disease-free survival (DFS) (P=0.013) of those with high ZEB1-AS1 expression decreased, indicating that abnormal expression of ZEB1-AS1 is an independent predictor of patient survival [133]. Another case demonstrated that high expression of MEG3 is associated with a good prognosis of HCC. Among the 72 HCC cases observed in this study, the DFS and OS of the group with high MEG3 expression were better than the group with low MEG3 expression, suggesting the potential of MEG3 expression level as an independent prognostic factor for HCC patients [134]. One study which has been done on 215 HCC patients found that high MVIH expression in cancer tissue was significantly associated with lower recurrence-free survival (RFS) and overall survival [135]. Moreover, high expression of MVIH is an independent risk factor for low RFS in patients.

Targeted therapy with lncRNAs also provides new ideas for disease treatment. In contrast to conventional therapies like chemotherapy, which impact both healthy and cancerous cells, targeted therapies are engineered to pinpoint and disrupt the precise disease-driving components, all while keeping harm to healthy cells at a minimum. Research on H19-based treatment methods has entered clinical trials. Under the dual control of H19 and IGF2-P4, a plasmid expressing diphtheria toxin can inhibit tumor growth, which has been confirmed in the ladder cancer [136], and it is expected to play a role in HCC with high expression of H19. Interference with related regulatory pathways by exogenous lncRNA is also a potential therapeutic approach. In a previous study, researchers used MS2 virus-like particles (VLPs) to cross-link with GE11 polypeptide and successfully constructed a MEG3 carrier, which was targeted to EGFR-positive HCC cells through the clathrin-mediated endocytosis [137]. The exogenously introduced MEG3 can inhibit the proliferation of HCC cells by enhancing the expression of p53 and downstream GDF15 and reducing the expression of MDM2. The results show the potential value of lncRNA combined with bioengineering in the treatment of HCC.

Recently, immunotherapy has become a hot spot in the field of cancer therapy. Playing important roles in immune regulation, lncRNAs show vast application value in the immunotherapy [138]. For example, lncEGFR can specifically bind to EGFR, and stabilize the expression of EGFR by blocking its interaction with c-CBL and subsequent ubiquitination, causing differentiation of regulatory T cells, inhibition of cytotoxic T cells, and progression of HCC [139]. Therefore, targeting and regulating lncRNA is highly potential for assisting immunotherapy and is worth further investigation in the future.

In recent years, there has been growing interest in the development of nanoparticle-based therapies for HCC that target lncRNAs. The most popular lncRNA for HCC nanoparticle therapy is MEG3 which acts as a tumor suppressor and regulates P53 target gene expression. However, lncRNA MEG3 demonstrates relatively low or no expression in human HCC. Therefore, researchers have been using MEG3 to suppress HCC cell proliferation, migration, invasion.

Ren et al. explored the use of polymeric nanoparticles for the targeted delivery of specific lncRNAs to HCC cells [140]. They utilized a polymer called pullulan, known for its efficient liver cell-targeting ability. This approach aimed to deliver plasmids encoding lncRNA MEG3 and the P53 gene. Co-delivery of these plasmids was achieved using a pullulan-based ethanolamine-modified poly(glycidyl methacrylate) (PuPGEA) polymer. This strategy effectively enhanced lncMEG3 and P53 expression in HCC cells, resulting in a stronger suppression of HCC cell proliferation, migration, invasion, and tumor growth. The study demonstrated the potential of these polymeric nanoparticles for dual-targeted theranostic gene delivery to HCC.

Later on, researchers [141] developed rod-like supramolecular nanoassemblies, denoted as CNC@CB[8]@PGEA, by combining various components, they are Cellulose Nanocrystals (CNCs), which are natural polysaccharide nanoparticles known for their biocompatibility and unique physicochemical properties, Poly(aspartic acid) Derivatives (PAsp), 1.Poly(cations) with Hydroxyl Groups (PGEA) and Cucurbit[8]uril (CB[8]). PAsp is used to enhance the disassembly and degradability of the nanoassemblies within cells. PGEA is a gene carrier with low cytotoxicity, attributed to its abundant hydroxyl groups. CB[8] is employed to facilitate host-guest interactions that play a crucial role in forming these supramolecular nanoassemblies. CNC@CB[8]@PGEA effectively complexes the expression constructs of two tumor-suppressive ncRNAs: miR-101 (plasmid pc3.0-miR-101) and lncRNA MEG3 (plasmid pc3.0-MEG3). Notably, this complex exhibits better transfection performance than assemblies containing PGEA alone. The key finding of this study is that the co-delivery system of CNC@CB[8]@PGEA, delivering both pc3.0-MEG3 and pc3.0-miR-101, demonstrates superior efficacy in suppressing HCC compared to using CNC@CB[8]@PGEA with either pc3.0-MEG3 or pc3.0-miR-101 alone. In summary, these rod-like supramolecular nanoassemblies hold promise as efficient delivery vectors for a range of tumor-suppressive nucleic acids, offering potential benefits for HCC treatment.

In another study, researchers [142] developed a novel therapeutic regimen with polymer nanoparticles to deliver lncRNA MEG3 to control the progression of HCC in a murine model. The study used Poly(lactic-co-glycolic acid) (PLGA) nanoparticles as delivery vehicles. The results indicated that administration of lncRNA MEG3-loaded nanoparticles led to improved histopathology and downregulation of tumor-associated markers, such as AFP, vascular endothelial growth factor (VEGF), and tumor necrosis factor-alpha (TNF-α). Furthermore, expression of markers like proliferating cell nuclear antigen (PCNA) and SUMO-specific protease 1 (SENP1) was reduced, and the expression of Caspase-3, an apoptosis marker, was increased. This demonstrated the potential of polymer nanoparticles as a novel therapeutic intervention for HCC via lncRNA delivery.

These studies showcase the versatility and effectiveness of polymer nanoparticles for the delivery of lncRNAs in HCC treatment. They highlight the potential of this approach in suppressing tumor growth and regulating gene expression in hepatocellular carcinoma. Further research and clinical trials are needed to assess the full therapeutic potential and safety of these nanoparticle-based lncRNA therapies for HCC.

The lncRNAs mentioned to be concerned with HCC diagnosis, prognosis and treatment are listed in Table 3. The lncRNA targets for HCC nanoparticle-based therapies are summarized in Table 4.

**Table 3:** Summary of LncRNAs that involved in the diagnosis, treatment, and prognosis of HCC.

**Aspect**	**Information**
**Diagnosis of HCC**	* Low early-stage diagnosis rate * AFP’s limited role as a diagnostic indicator * Emerging lncRNAs for diagnosis
	* UCA1: High specificity in screening HCC * UCA1 facilitates distinguishing benign liver disease from HCC
	* GAS5-AS1: Accurate in distinguishing HCC from cirrhosis * High sensitivity when AFP < 200 ng/ml
	* Other lncRNAs with diagnostic value: MALAT1, CASC9, SHNG1, Inc-PCDH9-13:1 * Combining lncRNAs for better diagnosis
**Prognosis of HCC**	- High HOTAIR expression linked to HCC recurrence - MALAT1 linked to high of HCC recurrence rate
	- ZEB1-AS1 impact on survival
	- MEG3 associated with better DFS and OS - High MVIH expression as an independent risk factor
**Targeted Therapy**	* H19-based treatment methods in clinical trials * Targeting H19 and IGF2-P4 for inhibiting tumor growth
	* Exogenous lncRNA introduction to interfere with regulatory pathways * MEG3 carrier construction
	* Immunotherapy involving lncRNAs: lncEGFR and immune regulation in HCC

**Table 4:** LncRNA targets for HCC nanoparticle-based therapies.

**Nanoparticle type**	**Targeted lncRNAs**	**Mechanism and findings**
Pullulan-based Poly(glycidyl methacrylate)	lncRNA MEG3, P53 gene	Enhanced lncRNA MEG3 and P53 expression, leading to stronger suppression of HCC cell proliferation, migration, invasion, and tumor growth.
Cellulose Nanocrystals (CNCs)	miR-101, lncRNA MEG3	CNC@CB[8]@PGEA effectively complexes the expression constructs of miR-101 and lncRNA MEG3 and the co-delivery system demonstrates superior efficacy in suppressing HCC
Poly(lactic-co-glycolic acid) (PLGA)	lncRNA MEG3	Markers such as AFP, VEGF, TNF-α, PCNA, and SENP1 were affected, and Caspase-3 expression was increased. It improved histopathology and downregulation of tumor-associated markers.

## Concluding remarks and perspectives

LncRNAs have crucial functions in regulating liver cancer progression in various ways, such as the occurrence, progression, metastasis, treatment, and prognosis of HCC (Figure 3). At present, researches on lncRNA in HCC mainly focus on single signaling pathways, while few of them systematically study the role of single lncRNA in multiple pathways to elucidate how it jointly regulates the progression, invasion, and metastasis of HCC. Although lncRNAs have shown promising effects on the diagnosis of HCC, there are still puzzles to be put together. The research on lncRNA is in its infancy, and its mechanism is still unclear. In addition, lncRNAs have numerous targets, and it is difficult to fully elucidate the regulatory network of a single lncRNA. The research on lncRNA is relatively scattered, and there is no systematic study on the correlation between specific lncRNA and HCC. A few studies have reported that lncRNAs have unique advantages in the diagnosis of HCC. However, the clinical studies of lncRNAs are still limited. In terms of the prognosis of lncRNAs, lots of studies have noticed the significant up- and down-regulation of lncRNA expression in HCC patients and proved that these changes are related to the prognosis of HCC. The specific mechanisms of how these lncRNAs affect the prognosis of HCC are still unrevealed. With the development of genetic techniques, lncRNA could become a general diagnosis method to observe the prognostic situation of diseases. For properly utilizing lncRNAs in the medical service, high-throughput sequencing methods and non-invasive detection methods also need to be created in the future studies. Invasion and metastasis of HCC is a relatively complicated process involving multi-factor interaction. Screening the crucial lncRNAs closely related to HCC metastasis requires a clear understanding of the biological functions and molecular mechanisms of lncRNAs. In addition, the in-depth exploration of the signaling pathways regulated by lncRNAs is also urgent to achieve a better understanding of the molecular mechanisms in the process of invasion and metastasis. Furthermore, clarifying the relationship between lncRNAs and the development of HCC is particularly vital for us to thoroughly understand the disease process and identify effective therapeutic targets and make better medical treatment strategies.
